# Obstetric outcomes in second-generation and migrant mothers: a cohort study in the Emilia-Romagna Region, Italy

**DOI:** 10.1007/s00404-025-08273-3

**Published:** 2026-03-31

**Authors:** Sara Cavagnis, Nicola Caranci, Enrica Perrone

**Affiliations:** 1 Department of Medical and Surgical Sciences, University of Bologna, 40100 Bologna, Italy; 2 Department of Innovation in Healthcare and Social Services, Emilia-Romagna Health and Welfare Directorate, Emilia-Romagna Region, 40127 Bologna, Italy; 3 Department of Community Care, Emilia-Romagna Health and Welfare Directorate, Emilia-Romagna Region, 40127 Bologna, Italy

**Keywords:** Migration, Health inequalities, Maternal health, Second generation

## Abstract

**Purpose:**

The second generation (people born in the host country to foreign parents) has grown significantly in Italy. While migration is known to impact maternal and neonatal health negatively, evidence on outcomes for second-generation mothers (SGMs) is limited. This study aimed to describe the socio-demographic characteristics and obstetric outcomes of SGMs, comparing them with first-generation and native mothers in an Italian region.

**Methods:**

All deliveries in the Emilia-Romagna Birth Registry (2012–2023) were linked with the 15th Population Census (2011); this allowed the definition of migratory background. The outcomes included emergency and elective cesarean delivery, preterm birth, small for gestational age (SGA), and late antenatal care (ANC). Log-binomial models were used to estimate crude and adjusted prevalence ratios (aPR), also stratified by area of origin.

**Results:**

390,062 births were recorded; for 63%, it was possible to define the migratory background. Compared to native mothers, SGMs were younger and less educated; 79.5% held Italian citizenship. SGMs showed a lower risk of elective cesarean delivery (aPR 0.65, 95% CI 0.53–0.79) and higher risks of late ANC (aPR 1.91, 95% CI 1.69–2.15), emergency cesarean delivery (aPR 1.15, 95%CI 1.00–1.31), preterm birth (aPR 1.59, 95% CI 1.33–1.90), and SGA (aPR 1.15, 95% CI 0.99–1.33). Certain areas of origin, such as Sub-Saharan Africa, seemed to have an increased risk of negative outcomes.

**Conclusions:**

SGMs show worse social determinants and birth outcomes than natives. Currently, the second generation cannot be identified through administrative data; however, it merits greater attention, in terms of monitoring tools and health and social policies.

**Supplementary Information:**

The online version contains supplementary material available at 10.1007/s00404-025-08273-3.

## What does this study add to the clinical work?

**Table:** 

This study shows that second-generation mothers, despite being born and raised in Italy, have higher risks of late antenatal care, emergency cesarean delivery, and preterm birth than native mothers. Clinicians should not assume equal obstetric risk based on citizenship or language proficiency and should proactively identify and support second-generation women throughout pregnancy.	

## Introduction

Migration represents an important determinant of health, with implications for both migrant mothers and their children. Migrants often face difficulties in accessing antenatal care (ANC) due to cultural and language barriers, limited knowledge of the healthcare system, and financial constraints [1, 2]. Such barriers can contribute to disparities in obstetric outcomes: migrant women may have higher rates of cesarean delivery, emergency cesarean delivery, preterm birth, and low birth weight compared to the native population [3–6]. They generally have a higher risk of severe maternal outcomes, including maternal and neonatal mortality [7]. These risks vary according to both the host country’s characteristics (income, continent, migration and integration policies) and the mother’s country of origin [5, 7].

Second-generation individuals are those born in and residing in a country that their parents previously entered as migrants. While definitions of second generation can vary, they generally refer to individuals born in the host country to foreign-born parents, though in some cases, individuals with a mixed background (where only one parent is a migrant) or those who arrived at a very young age may also be included [8, 9]. In the European Union, as of 2023, 3.0% of individuals aged 15–74 were native-born with two foreign-born parents; in Italy, this figure was lower at 0.9% [10]. The country has a relatively recent immigration history, and the majority of the second generation would be younger than 15: the number of second-generation adults is expected to increase.

Research on the health outcomes of the second generation, especially in Europe, is limited. A review showed that second generations in Europe, particularly those with non-European origins, have higher early life and adult mortality risks than natives [11]. Results from the European Social Survey show that this population had the highest prevalence of poor self-reported health compared to both migrants and natives; inequalities were wider for women with lower socio-economic status [12]. This trend is particularly relevant in light of the *healthy immigrant effect*, according to which international migrants have lower mortality rates than natives [13]. Worse health among second-generation people could signal challenges in migrant integration and anti-discrimination policies. This inter-generational worsening of health has also been described in a systematic review on birth weight, showing that second-generation mothers experience an increased risk of having a low birth-weight child compared to migrants [14]. Most evidence, however, comes from the USA and UK: there is little evidence on the obstetric outcomes of the second generation in Europe.

Given the growing second generation in Italy, it is crucial to understand how this group performs regarding obstetric outcomes and to identify the factors that may contribute to disparities. This study aimed to describe the socio-demographic characteristics and obstetric outcomes of second-generation mothers in an Italian region and to compare these outcomes with those of native Italians. We hypothesized that having a migratory background would influence the outcomes for second-generation mothers, although to a lesser extent than for migrants.

## Methods

### Study design and data sources

This retrospective cohort study included all singleton pregnancies in the Emilia-Romagna region between 01/01/2012 and 31/12/2023. Emilia-Romagna is a region in north-eastern Italy with a population of 4.4 million residents and a high proportion of migrants. All residents are offered ANC and obstetric care free of charge, through the National Health Service (NHS); in Emilia-Romagna, the proportion of women using a public provider for ANC has increased over time, reaching 67% in 2023, while around a third chose a private provider. Antenatal classes are offered by both the NHS and private entities.

The data source was the regional Birth Registry (CedAP, Certificato di Assistenza al Parto), which is filled in at birth for each delivery by a midwife or a doctor. CeDAP contains data on pregnancy, delivery, the mother, and the newborn. Mothers in CedAP are identified by a numerical code used in all regional databases to comply with the national privacy legislation. This code was used to link birth data to the 2011 Italian Census to define the mothers' migratory background.

## Measures: exposure, outcomes and covariates

The main exposure was migratory background, which was based on the country of birth of the mother and her parents as recorded in the Census. Women born in Italy with both parents born abroad were defined as second generation; those born abroad from both parents born abroad were first-generation migrants. Women born in Italy to parents born in Italy, as well as women born in Italy to one Italian-born and one foreign-born parent (whose sociodemographic characteristics and outcomes were similar in preliminary analyses), were classified as natives.

Area of origin was defined as the country of birth for the first generation and the parents’ country of birth for the second generation. If parents had different countries of birth, the mother’s was used. Countries were grouped into geographical regions; Eastern Europe was analysed separately from the EU and other Western countries due to its distinct migration history (see **Supplementary materials** for regions’ definition).

Four main outcomes were investigated: late ANC (first visit after 11^+6^ weeks of gestational age—GA), cesarean delivery (overall, elective and emergency), preterm birth (birth before 37 weeks of GA), and small for gestational age (SGA, weight at birth < 10th percentile for GA). As a secondary outcome, we included very preterm birth (< 32 weeks of GA).

Socio-demographic covariates included maternal age, education, occupation, and citizenship, which were recorded in the Birth Registry. Education was categorized as low (middle school or less), medium (high school), or high (university); occupation was grouped into employed, unemployed, housewife, and student/other. Citizenship was dichotomized into Italian and foreign.

Other relevant covariates included parity (0 and ≥ 1 previous birth), assisted reproductive technology (ART), and antenatal class attendance. We included the period of delivery (2012–14, 2015–2017, 2018–2020, and 2021–2023) to account for trends in the outcomes.

### Analysis

After detailing the characteristics of the patients, we employed log-binomial models to estimate crude and adjusted prevalence ratios (aPR), with a 95% confidence interval (CI), for the association between migratory background and outcomes. For the cesarean delivery outcomes we used a Poisson model with robust standard errors due to convergence issues of the log-binomial model. The reference group consisted of natives. We adjusted the models for maternal age in class (< 25, 25–29, 30–34, 35–39, 40–44, and > 44 years), education, occupation, parity, ART, and time period. As education level and occupation could lie on the causal pathway between migration background and outcomes, we also ran a sensitivity model excluding these variables while retaining other covariates. In a secondary analysis, we estimated aPRs for the area of origin, stratifying by generation and adjusting for maternal age, education, occupation, parity, and time period; this analysis was limited to emergency cesarean delivery, preterm birth, and SGA. Data analysis was conducted using Stata SE v17.0.

### Ethics

CedAP is mandatory by law [15] and is collected by the Health Information Systems of Emilia-Romagna Region [16] to assess the appropriateness, effectiveness and efficiency of the healthcare provided. The study is nested within the study “Studio Longitudinale dell’Emilia-Romagna”, which is included in the Italian Statistical Program [17]; privacy and ethical issues have been assessed by the Italian Data Protection Authority (Institutional Board: Italian Data Protection Authority; ID EMR-00019).

## Results

Between 2012 and 2023, 390,062 singleton deliveries were registered in Emilia-Romagna; 258,597 were linked to the Census. We were able to define the mother’s migratory background for 246,521 deliveries (63% of all deliveries in the period); these constitute the final study population, as described in Table 1.

**Table 1:** Socio-demographic and clinical characteristics of the study population, by migratory background

	Natives	Second generation	First generation
N (row %)	191,803 (77.8%)	1,720 (0.7%)	52,998 (21.5%)
Age at delivery (mean, SD)	33.3 (5.1)	26.2 (5.5)	31.4 (5.4)
Education level (%)			
High	40.6	14.3	15.3
Medium	45.1	52.6	42.3
Low	14.3	33.1	42.4
Occupation (%)			
Employed	84.0	49.1	44.7
Unemployed	6.6	16.6	8.6
Housewife	8.3	28.1	45.5
Student or other	1.1	6.2	1.2
Italian citizenship (%)	99.7	79.5	14.5
Main areas of origin (%)	–	Northern Africa (32.6%), Eastern Europe (18.3%), Eastern Asia (14.7%)	Eastern Europe (26.1%), Northern Africa (25.4%), EU and other Western countries (16.6%)
Multiparous (%)	45.2	38.0	68.1
Use of ART (%)	3.1	1.0	1.6
Antenatal class attendance (%)	61.6	35.7	21.7

77.8% of the population were natives, followed by first-generation (21.5%); second-generation mothers comprised a small proportion of all mothers (0.7%, n = 1,720). The percentage of second-generation mothers increased in the period considered, from 0.3% of all deliveries in 2012 to 1.7% in 2023.

Second-generation mothers, compared to natives, were younger (mean age 26.2 vs 33.3), had lower education, and were less often employed. These characteristics are similar, to a smaller extent, to those of first-generation migrants. Second-generation mothers mainly originated from Northern Africa, Eastern Europe, and Eastern Asia. While most migrants had a previous delivery (68.1%), second-generation mothers and natives were more often nulliparous (62.0% and 54.8%, respectively). Pregnancies following ART were more common in natives. Natives also attended antenatal classes more often than second-generation mothers (61.6% vs 35.7%).

Both first- and second-generation mothers had a higher risk of accessing ANC late (aPRs, respectively, 1.91 (95%CI 1.69–2.15) and 1.99 (95%CI 1.93–2.06)) (Table 2).

**Table 2:** Association between migration background and the obstetric outcomes

	Cases (row %)	Crude PR (95% CI)	aPR (95% CI) *	*p* value
**Late ANC**				
Natives	10,368 (5.4)	1	1	
Second generation	245 (14.3)	2.64 (2.35–2.97)	1.91 (1.69–2.15)	< 0.001
First generation	8,615 (16.3)	3.01 (2.93–3.09)	1.99 (1.93–2.06)	< 0.001
**Cesarean delivery**				
Natives	47,411 (24.7)	1	1	
Second generation	295 (17.1)	0.69 (0.62–0.77)	0.91 (0.82–1.01)	0.084
First generation	13,214 (24.9)	1.01 (0.99–1.03)	1.02 (1.00–1.04)	0.066
**Elective cesarean delivery**				
Natives	24,368 (12.7)	1	1	
Second generation	100 (5.8)	0.46 (0.38–0.55)	0.65 (0.53–0.79)	< 0.001
First generation	7,169 (13.5)	1.06 (1.04–1.09)	0.92 (0.89–0.94)	< 0.001
**Emergency cesarean delivery**				
Natives	23,043 (12.0)	1	1	
Second generation	195 (11.3)	0.94 (0.83–1.08)	1.15 (1.00–1.31)	0.049
First generation	6,045 (11.4)	0.95 (0.92–0.97)	1.16 (1.12–1.19)	< 0.001
**Preterm birth**				
Natives	10,063 (5.3)	1	1	
Second generation	122 (7.1)	1.35 (1.14–1.61)	1.59 (1.33–1.90)	< 0.001
First generation	3,460 (6.5)	1.24 (1.20–1.29)	1.27 (1.21–1.32)	< 0.001
**Very preterm birth**				
Natives	1,309 (0.7)	1	1	
Second generation	11 (0.6)	0.94 (0.52–1.69)	1.10 (0.60–2.00)	0.765
First generation	572 (1.1)	1.58 (1.43–1.74)	1.63 (1.45–1.83)	< 0.001
**SGA**				
Natives	15,540 (8.1)	1	1	
Second generation	165 (9.6)	1.18 (1.02–1.37)	1.15 (0.99–1.33)	0.069
First generation	4,059 (7.7)	0.94 (0.91–0.98)	0.85 (0.82–0.88)	< 0.001

*Adjusted for maternal age in class, maternal education level, occupation, parity, ART, and period

Second-generation mothers did not have a significant difference in overall cesarean delivery rate compared to natives (aPR 0.91, 95% CI 0.82–1.01), while migrants had a slightly increased risk. On the other hand, both second-generation mothers and migrants had a lower risk of elective cesarean delivery than natives (respectively, aPR 0.65, 95%CI 0.53–0.79 and 0.92, 95%CI 0.89–0.94). Regarding emergency cesarean deliveries, both second- and first-generation mothers had a higher risk than natives (respectively, aPR 1.15 (95%CI 1.00–1.31) and 1.16 (95%CI 1.12–1.19)).

The risk of preterm birth was higher in mothers with a migratory background than in natives, particularly for second-generation mothers (aPR 1.59, 95%CI 1.33–1.90). Migrants had a higher risk than natives of very preterm birth (aPR 1.63, 95%CI 1.45–1.83), while the risk did not differ between natives and the second generation.

Second-generation mothers also had a higher risk of SGA than natives (aPR 1.15, 95%CI 0.99–1.33), while immigrants had a lower risk (aPR 0.85, 95%CI 0.82–0.88).

Estimates from partially adjusted models were consistent with those of the main model in terms of the direction of association, although slight differences in magnitude were observed (see **Table S2** in Supplementary materials).

Table 3 describes the association between area of origin and selected outcomes, by generation. Regarding emergency cesarean delivery, while first-generation mothers overall had a higher risk than natives, migrants from some areas had a higher risk (Sub-Saharan Africa, Central and Southern America, and Western, Central and Southern Asia), while migrants coming from the EU, Eastern Europe, and Eastern Asia had a risk comparable to natives. Second-generation mothers whose parents migrated from Western, Central and Southern Asia had a higher risk of emergency cesarean delivery than natives.

**Table 3:** Association between area of origin and emergency cesarean delivery, preterm birth and SGA, by migration background, compared to natives (aPR (95% CI))

	Emergency cesarean delivery		Preterm birth		SGA	
	First generation	Second generation	First generation	Second generation	First generation	Second generation
EU and other Western countries	0.99 (0.93–1.05)	0.83 (0.51–1.35)	1.34 (1.24–1.45)	0.93 (0.45–1.92)	0.77 (0.71–0.83)	0.86 (0.49–1.52)
Eastern Europe	1.02 (0.96–1.07)	1.30 (0.96–1.75)	1.09 (1.01–1.18)	1.41 (0.92–2.16)	0.71 (0.66–0.76)	1.16 (0.82–1.63)
Northern Africa	1.06 (1.00–1.13)	1.16 (0.92–1.47)	1.07 (0.99–1.16)	1.43 (1.04–1.97)	0.72 (0.67–0.78)	1.03 (0.79–1.36)
Sub-Saharan Africa	1.92 (1.81–2.04)	1.29 (0.88–1.89)	1.66 (1.52–1.82)	1.66 (1.00–2.76)	1.07 (0.98–1.16)	2.02 (1.45–2.80)
Western, central and southern Asia	1.36 (1.26–1.47)	1.65 (1.03–2.62)	1.53 (1.38–1.70)	1.36 (0.63–2.95)	1.64 (1.52–1.76)	1.88 (1.16–3.03)
Eastern Asia	1.01 (0.90–1.13)	0.79 (0.52–1.22)	1.41 (1.22–1.62)	1.68 (1.09–2.59)	0.84 (0.74–0.97)	1.14 (0.77–1.69)
Central and southern America	1.37 (1.26–1.48)	1.20 (0.72–2.00)	1.36 (1.20–1.54)	2.21 (1.26–3.86)	0.78 (0.69–0.89)	0.69 (0.32–1.50)

*Adjusted for maternal age in class, maternal education level, occupation, parity, and period

The estimated risk of preterm birth was particularly high for mothers originating from Sub-Saharan Africa (aPR 1.66 for both first and second generations) and for second-generation mothers from Central and Southern America (aPR 2.21, 95%CI 1.26–3.86) and Eastern Asia (aPR 1.68, 95%CI 1.09–2.59).

First-generation mothers from Western, Central and Southern Asia had an increased risk of SGA (aPR 1.64, 95%CI 1.52–1.76), differently from those from other areas of origin. The second generation originating from Sub-Saharan Africa and Western, Central and Southern Asia had an increased risk of SGA (respectively, aPR 2.02 and 1.88).

## Discussion

This study shows that second-generation mothers have a peculiar pattern of obstetric outcomes. They showed social disadvantage characteristics comparable to migrants: only half were employed (compared to 84% of native women), and 33% had a low level of education, more than double that of natives. Similar to migrants, second-generation mothers had a higher risk of late ANC, emergency cesarean delivery and preterm birth than natives. On the other hand, they had a higher chance than natives of having a SGA newborn (although this result was not significant), while migrants have a lower risk. There was substantial heterogeneity according to the area of origin, with some regions showing consistent evidence of worse health outcomes (Sub-Saharan Africa, Western, Central and Southern Asia).

This study has several strengths. To the best of our knowledge, this is the first study of obstetric outcomes of second-generation mothers in Italy, and among the few conducted in Europe. It covers a long period of time in a large Italian region; the Birth Registry is analysed annually and the results are discussed with healthcare professionals to improve its quality and validity. The linkage with the Census allowed us to accurately define the mothers’ migration background, since electronic health records only include country of birth and citizenship, which cannot identify the second generation, and ethnicity is currently not recorded in Italy.

Alongside the strengths discussed above, a number of limitations need to be acknowledged. Using the Census means we had to exclude from the analyses all mothers not living in the Region in 2011: the results for first-generation migrants cannot be generalized to all migrants in the period considered, since the region experienced a high influx in 2015–2016. However, the included population likely represents more stable migrants, who should have had better access to healthcare and welfare services. Sensitivity analyses confirm this hypothesis, showing that non-linked mothers less frequently had Italian citizenship, were less employed, and had lower education; the distribution of the obstetric outcomes was similar between linked and non-linked deliveries (Tables S3 and S4 in Supplementary materials). Another limitation is that the immigration history in Italy is relatively recent: second-generation women have not yet experienced the full span of reproductive life. Therefore, our results should be considered partial and may change with the consolidation of this population. In addition, the analyses stratified by area have a low number of subjects in some groups: the lack of significant differences might be explained by low power. We did not have information on the size or volume of the maternity clinic in our data set; institutional factors of this kind may influence obstetric outcomes and should be considered in future research. Finally, we could not adjust for some behavioural covariates (e.g., smoking habits or alcohol consumption), which are not present in CeDAP or are recorded differently between health facilities.

There was no significant difference in terms of overall cesarean delivery rates between second-generation mothers and native mothers. However, this study showed that second-generation mothers have a higher risk of delivering via emergency cesarean delivery and a lower risk of elective cesarean delivery. Emergency cesarean delivery could be more frequent when ANC is accessed late or inappropriately, and maternal or foetal disorders are not diagnosed in time to plan an elective cesarean delivery [18]. The lower rate of elective cesarean delivery could also relate to the higher proportion of primiparas among second-generation mothers, who are less likely to have a previous cesarean delivery—one of the main indications for elective procedures. This association remained after adjusting for parity in the analysis, suggesting that other factors may also contribute. Disparities in planned obstetric interventions are also documented in other contexts: a recent scoping review reported that ethnic minority women had reduced access to elective induction of labor despite comparable clinical indications [19]. These differences in planned care mirror our findings on elective cesarean delivery and may reflect unequal access to anticipatory or preference-sensitive obstetric decision-making. Two studies conducted in Germany reported no significant differences in emergency cesarean delivery rates compared to native mothers [20, 21]. In both studies, most second-generation mothers were Turkish or Lebanese: different areas of origin could explain the difference with our findings, along with differing integration policies and cesarean delivery practices. Our result of a lower risk of elective cesarean delivery is consistent with findings from Germany and Norway [21–23].

Preterm birth was more frequent among mothers with a migratory background compared to natives, with a higher risk increase for second-generation mothers than for migrants. Other European studies on the risk of preterm birth in second-generation mothers show inconclusive results [22–24]. According to a recent Italian study, ANC adherence mediates the higher risk of preterm birth in migrants, and improving ANC might reduce preterm birth rates by up to 37% [25]. In this study, second-generation mothers accessed healthcare services later and attended antenatal classes less frequently; this might compromise the ability to provide timely information and preventive interventions and to identify any pathological conditions. While limited language proficiency may influence ANC access among first-generation migrants, it is unlikely to explain these patterns in second-generation mothers, who are most likely fluent. This underscores the importance of identifying barriers and determinants of healthcare access among second-generation mothers, to facilitate interventions aimed at reducing disparities.

There was a significant difference according to migration background regarding SGA: second-generation mothers had SGA newborns more often than natives, while migrants seemed to have a lower risk. The anthropometric charts currently in use in Emilia-Romagna were validated for the Italian population in 2010 [26]. Newborns of specific ethnic groups can be both significantly heavier and lighter than Italian babies [27], and growth may be influenced by genetic and epigenetic factors related to ethnicity, making the observed difference in birth weight physiological [28, 29]. The difference between first- and second-generation mothers in this study may, therefore, be explained by the distinct composition of the two groups regarding area of origin. This is supported by the analysis stratified by area of origin: both generations from the same area had a similar risk of SGA (e.g., mothers from Central, Southern, and Western Asia had a higher risk of SGA in both generation). Conversely, the difference in risk could be attributed to variations in behaviours during pregnancy and barriers to accessing healthcare services [30], potentially leading to poorer health outcomes; a systematic review showed that children of second-generation mothers have a higher risk of low birth weight compared to children of immigrant mothers [14]. Our results on SGA should be interpreted with caution and warrant further investigation into the appropriateness of current growth charts and the health outcomes of SGA babies by area of origin.

The health trajectories observed in SGMs are influenced by complex factors. On one hand, according to the process defined as “negative acculturation”, mothers from non-Italian backgrounds may adopt unhealthy lifestyles that are frequent in the host country, such as smoking or poor diet. On the other hand, the worsening health of immigrants and their descendants may be due to other structural forces [31]. Despite similar education and income, foreign-origin populations often face barriers, compared to natives, to quality employment or housing, perpetuating inequality [32]. According to a recent systematic review, living in socioeconomically deprived areas is associated with significantly worse perinatal outcomes [33]. The weathering hypothesis suggests that health deteriorates gradually due to chronic exposure to stress factors, such as social inequalities and racial discrimination [34]. As individuals age, these effects accumulate, influencing both general and reproductive health, with transgenerational effects. While this study does not measure ethnicity or experience of discrimination, previous research in Italy and Europe has documented the association between ethnic discrimination and poorer physical and mental health [35–37]. Many migrant populations in Italy belong to racialized minority groups, which may intersect with social disadvantage and structural barriers, contributing to differences in obstetric outcomes. Further research, in Italy and across Europe, is needed to describe the effects of migration background and racism, particularly as a structural phenomenon, on living conditions and health, to develop strategies to counter inequalities [38].

In the Emilia-Romagna region, the disadvantages faced by women born abroad have persisted in the second generation. The healthcare system should be more effective in promoting health throughout life, beginning from childhood. Antenatal and obstetric care in Emilia-Romagna is based on evidence-based maternity care models, such as specialist multidisciplinary teams; however, some important features, including culturally competent healthcare providers, could be strengthened to improve outcomes [39].

While our original hypothesis was that second-generation mothers would be in-between migrants and natives, they are actually closer to migrants in terms of negative health outcomes. This study's insights and findings emphasize the years before pregnancy as an ideal time to implement welfare policies, enhance inclusion and anti-discrimination efforts, and provide educational support, to break the transgenerational chain of disadvantage. This is critical, because the number of second-generation mothers, in Italy and in Europe, will increase. Greater attention is needed in research, monitoring, and planning of welfare and healthcare policies and services for second-generation mothers.

## Supplementary Information

Below is the link to the electronic supplementary material.

Supplementary file1 (DOCX 18 KB)

## Data Availability

The data used in this study cannot be made publicly available due to Italian data protection laws.
