# Supplementary material for: Obstetric outcomes in second-generation and migrant mothers: a cohort study in the Emilia-Romagna Region, Italy
Source: Arch Gynecol Obstet. 2026 Mar 31;313(1):148. doi: 10.1007/s00404-025-08273-3 (PMC13038752; doi:10.1007/s00404-025-08273-3)
Supplement: Supplementary file 1 — Supplementary file1 (DOCX 18 KB) [file 404_2025_8273_MOESM1_ESM.docx]

**Supplementary materials**

| Table S1. Definition of areas of origin | |  |
| --- | --- | --- |
| EU and other Western countries | EU15 (Austria, Belgium, Denmark, Finland, France, Germany, Greece, Ireland, Luxembourg, Netherlands, Portugal, United Kingdom, Spain, Sweden), Other European Countries (Andorra, Iceland, Liechtenstein, Malta, Monaco, Norway, San Marino, Switzerland, Vatican City), North America (Canada, USA), Oceania (Australia, Federated States of Micronesia, Fiji, Kiribati, Marshall Islands, Nauru, New Zealand, Palau, Papua New Guinea, Samoa, Solomon Islands, Tonga, Tuvalu, Vanuatu), Israel, Japan | |
| Eastern Europe | Albania, Belarus, Bosnia and Herzegovina, Bulgaria, Croatia, Cyprus, Estonia, Latvia, Lithuania, North Macedonia, Moldova, Poland, Czech Republic, Romania, Russia, Serbia, Montenegro, Slovakia, Slovenia, Turkey, Ukraine, Hungary | |
| Northern Africa | Algeria, Egypt, Libya, Morocco, South Sudan, Sudan, Tunisia | |
| Sub-Saharan Africa | Angola, Benin, Botswana, Burkina Faso, Burundi, Cameroon, Cape Verde, Central African Republic, Chad, Comoros, Congo, Democratic Republic of the Congo, Djibouti, Equatorial Guinea, Eritrea, Ethiopia, Gabon, Gambia, Ghana, Guinea, Guinea-Bissau, Ivory Coast, Kenya, Lesotho, Liberia, Madagascar, Malawi, Mali, Mauritania, Mauritius, Mozambique, Namibia, Niger, Nigeria, Rwanda, São Tomé and Príncipe, Senegal, Seychelles, Sierra Leone, Somalia, South Africa, eSwatini, Tanzania, Togo, Uganda, Zambia, Zimbabwe | |
| Western, central and southern Asia | Afghanistan, Armenia, Azerbaijan, Bahrain, Bangladesh, Bhutan, Georgia, India, Iran, Iraq, Jordan, Kazakhstan, Kuwait, Kyrgyzstan, Lebanon, Maldives, Nepal, Oman, Pakistan, Qatar, Saudi Arabia, Sri Lanka, Syria, Tajikistan, Palestinian Occupied Territories, Turkmenistan, United Arab Emirates, Uzbekistan, Yemen | |
| Eastern Asia | Myanmar (formerly Burma), Brunei, Cambodia, China, North Korea, South Korea, Philippines, Indonesia, Laos, East Timor, Malaysia, Mongolia, Singapore, Thailand, Vietnam, Taiwan | |
| Central and southern America | Antigua and Barbuda, Argentina, Bahamas, Barbados, Belize, Bolivia, Brazil, Chile, Colombia, Costa Rica, Cuba, Dominica, Ecuador, El Salvador, Grenada, Jamaica, Guatemala, Guyana, Haiti, Honduras, Mexico, Nicaragua, Panama, Paraguay, Peru, Dominican Republic, Saint Kitts and Nevis, Saint Lucia, Saint Vincent and the Grenadines, Suriname, Trinidad and Tobago, Uruguay, Venezuela | |

| Table S2. Association between migration background and the obstetric outcomes, intermediate model (not adjusted for occupation and education level) | | | | |
| --- | --- | --- | --- | --- |
|  | Cases (row %) | Crude PR (95% CI) | aPR (95% CI) | p value |
| **Late ANC** | | | | |
| Natives | 10,368 (5.4) | 1 | 1 |  |
| Second generation | 245 (14.3) | 2.64 (2.35-2.97) | 2.28 (2.02-2.56) | <0.001 |
| First generation | 8,615 (16.3) | 3.01 (2.93-3.09) | 2.53 (2.46-2.61) | <0.001 |
| **Cesarean delivery** | | | | |
| Natives | 47,411 (24.7) | 1 | 1 |  |
| Second generation | 295 (17.1) | 0.69 (0.62-0.77) | 0.96 (0.86-1.06) | 0.408 |
| First generation | 13,214 (24.9) | 1.01 (0.99-1.03) | 1.09 (1.08-1.11) | <0.001 |
| **Elective cesarean delivery** | | | | |
| Natives | 24,368 (12.7) | 1 | 1 |  |
| Second generation | 100 (5.8) | 0.46 (0.38-0.55) | 0.70 (0.58-0.85) | <0.001 |
| First generation | 7,169 (13.5) | 1.06 (1.04-1.09) | 1.00 (0.98-1.03) | 0.878 |
| **Emergency cesarean delivery** | | | | |
| Natives | 23,043 (12.0) | 1 | 1 |  |
| Second generation | 195 (11.3) | 0.94 (0.83-1.08) | 1.19 (1.04-1.36) | 0.012 |
| First generation | 6,045 (11.4) | 0.95 (0.92-0.97) | 1.23 (1.19-1.26) | <0.001 |
| **Preterm birth** | | | | |
| Natives | 10,063 (5.3) | 1 | 1 |  |
| Second generation | 122 (7.1) | 1.35 (1.14-1.61) | 1.66 (1.39-1.98) | <0.001 |
| First generation | 3,460 (6.5) | 1.24 (1.20-1.29) | 1.40 (1.34-1.45) | <0.001 |
| **Very preterm birth** | | | | |
| Natives | 1,309 (0.7) | 1 | 1 |  |
| Second generation | 11 (0.6) | 0.94 (0.52-1.69) | 1.11 (0.61-2.02) | 0.733 |
| First generation | 572 (1.1) | 1.58 (1.43-1.74) | 1.84 (1.66-2.04) | <0.001 |
| **SGA** | | | | |
| Natives | 15,540 (8.1) | 1 | 1 |  |
| Second generation | 165 (9.6) | 1.18 (1.02-1.37) | 1.21 (1.04-1.40) | 0.012 |
| First generation | 4,059 (7.7) | 0.94 (0.91-0.98) | 0.96 (0.93-1.00) | 0.037 |

| Table S3. Socio-demographic and clinical characteristics comparing included and excluded deliveries | | |
| --- | --- | --- |
|  | Excluded | Included |
| N (row %) | 143541 (37%) | 246521 (63%) |
| Age at delivery (mean, SD) | 30,8 (5,7) | 32,8 (5,2) |
| Education level |  |  |
| High (%) | 31% | 35% |
| Medium (%) | 37% | 45% |
| Low (%) | 32% | 20% |
| Occupation |  |  |
| Employed (%) | 47% | 75% |
| Unemployed (%) | 11% | 7% |
| Housewife (%) | 41% | 16% |
| Student or other (%) | 1% | 1% |
| Citizenship (% Italian) | 46% | 81% |
| Multiparous (%) | 48% | 50% |
| Use of ART (%) | 1.8% | 2.8% |
| Late ANC (%) | 14% | 8% |

| Table S4. Distribution of outcomes comparing included and excluded deliveries | | |
| --- | --- | --- |
|  | Excluded | Included |
| N (row %) | 143541 (37%) | 246521 (63%) |
| Preterm birth (%) | 5.9% | 5.5% |
| Very preterm birth (%) | 1.0% | 0.8% |
| Emergency c-section (%) | 12.2% | 11.9% |
| Elective c-section (%) | 11.5% | 12.8% |
| SGA (%) | 8.6% | 8.0% |
